# Current Insights: The Impact of Gut Microbiota on Postoperative Complications in Visceral Surgery—A Narrative Review

**DOI:** 10.3390/diagnostics11112099

**Published:** 2021-11-13

**Authors:** Ann-Kathrin Lederer, Sophia Chikhladze, Eva Kohnert, Roman Huber, Alexander Müller

**Affiliations:** 1 Center for Complementary Medicine, Department of Medicine II, Medical Center—University of Freiburg, Faculty of Medicine, University of Freiburg, 79106 Freiburg, Germany; roman.huber@uniklinik-freiburg.de (R.H.); alexander.mueller@uniklinik-freiburg.de (A.M.); 2 Chirurgische Klinik, Evangelisches Diakoniekrankenhaus Freiburg, 79106 Freiburg, Germany; 3 Department of General and Visceral Surgery, Medical Center—University of Freiburg, 79106 Freiburg, Germany; sophia.chikhladze@uniklinik-freiburg.de; 4 Institute of Medical Biometry and Statistics, Medical Center—University of Freiburg, Faculty of Medicine, University of Freiburg, 79104 Freiburg, Germany; kohnert@imbi.uni-freiburg.de

**Keywords:** postoperative complications, outcome, gut microbiome, anastomotic leakage, surgical site infections, ileus

## Abstract

Postoperative complications are a major problem occurring in up to 50% of patients undergoing major abdominal surgery. Occurrence of postoperative complications is associated with a significantly higher morbidity and mortality in affected patients. The most common postoperative complications are caused by an infectious genesis and include anastomotic leakage in case of gastrointestinal anastomosis and surgical site infections. Recent research highlighted the importance of gut microbiota in health and disease. It is plausible that the gut microbiota also plays a pivotal role in the development of postoperative complications. This narrative review critically summarizes results of recent research in this particular field. The review evaluates the role of gut microbiota alteration in postoperative complications, including postoperative ileus, anastomotic leakage, and surgical site infections in visceral surgery. We tried to put a special focus on a potential diagnostic value of pre- and post-operative gut microbiota sampling showing that recent data are inhomogeneous to identify a high-risk microbial profile for development of postoperative complications.

## 1. Introduction

Postoperative complications are a serious but so far largely unpreventable burden in visceral surgery, leading to a higher morbidity and mortality rate in affected patients [1,2,3,4]. The occurrence of postoperative complications is in addition a significant economic burden for healthcare systems [2,5,6]. Major complications are rare in case of minor surgery in healthy patients, but occur in up to 50% of patients undergoing complex visceral surgery such as pancreas, liver or esophagus surgery [7,8,9].

The most common surgical postoperative complications are caused by infections including surgical site infections (SSI) as well as surgery-depending complications such as anastomotic leakage (AL) and secondary infected pancreatic fistula [3,10]. The occurrence of an infectious complication is often patient-related as patients’ microbial colonization is suggested to be the main source of infection [2,11,12,13]. Moreover, in critical illness, the gut may serve as an infectious source due to bacterial translocation leading to a systemic inflammatory response and even sepsis [14]. The human gastrointestinal tract is the residence of the so called gut microbiome, which can be modulated by several internal and external factors [15,16]. The gut microbiome is the totality of all gastrointestinal microorganisms (bacteria, viruses, protozoa, and fungi) and their collective genetic material. It has to be differentiated from the term “microbiota”, which refers to microorganisms that are found in a specific environment such as the human body. Recent studies suggest that the gut microbiota plays an important role in the development of cardiovascular diseases, obesity, cancer and bowel diseases [17,18,19]. Thus, it is conceivable that the gut microbiota also affects surgical outcomes. 

In general, strategies to prevent postoperative complications due to patients’ microbial colonization include sufficient preoperative antiseptic preparation of skin and intraoperative antimicrobial prophylaxis as well as preoperative gut decontamination in case of gastrointestinal anastomosis [12,13,20]. Typical pathogens causing infectious complications are often beneficial commensals of the human body living in an equilibrium with the host. The kind of pathogen depends on the type of surgery and location of infection, but the most common bacteria leading to postoperative infections are *Staphylococcus*, *Enterococcus, Bacteroides* and *Escherichia coli* [21,22]. The existence of these inhabitants is essential for the function of the immune system, for providing digestion of foods and nutrient processing in a healthy human being [23]. Furthermore, recent animal studies suggests an impact of gut microbiota on the gut sensorimotor function, which is relevant for postoperative recovery of the bowel function [24]. A postoperative paralysis of the gastrointestinal tract is the most common non-infectious complication after surgery. Clinical features and symptoms include abdominal pain, feeling of fullness, nausea, and vomiting. Postoperative intestinal paralysis is associated with a higher postoperative morbidity and a longer hospital stay [25].

Still to date, it is widely unclear, why some patients develop complications and others do not, even though cases apparently share common clinical features. In the last decade, the hypothesis of an involvement of the gut microbiota composition for development of postoperative complications has been suggested. It appears that complications are not caused just by single bacteria as surgery leads to a bacterial adaption and selection of all gut microorganisms [26]. The composition of the gut microbiota is a sensitive, partially self-regulating, and self-controlling equilibrium with commensal bacteria being able to target and kill intestinal pathogens [22,27,28].

In 2017, our research group performed a systematic review to clarify, if postoperative complications are related to the patients’ gut microbiota [29]. We found some potential relationship between the gut microbiota and the development of postoperative complications, but we were unable to draw firm conclusions due to several methodological flaws of included studies. This time, we decided to evaluate recent research results by a narrative review to give a broad overview of data. In light of the aforementioned findings, the relevance of patients’ gut microbiota composition for the postoperative outcome in visceral surgery is highly suggested by recent research. This narrative review sought to summarize recent studies evaluating the relation between gut microbiota and postoperative complications in visceral surgery. We put a special focus on a potential diagnostic value of pre- and post-operative gut microbiota sampling to detect high risk patients and to prevent postoperative complications.

## 2. Methods

We performed a narrative literature review between July and August 2021. All kind of studies, whether human or animal trials, were eligible for evaluation. There was no limitation due to age or gender of patients and all surgical procedures of the gastrointestinal tract with except of bariatric surgery were considered. Databases of Medline, the Cochrane Library and Google Scholar were searched to review publications. Search terms were “gut microbiome/microbiota”, “surgical site infection”, “anastomotic leakage”, “ileus” and “postoperative complications”. In addition, reference lists of included studies and reviews were screened by title and abstract for eligible publications. Abstracts and manuscripts were evaluated in English, German, Italian, French and Spanish. No further search restrictions were applied. To ensure quality of this narrative review, manuscript was prepared according to the Scale for the Assessment of Narrative Review Articles (SANRA) [30]. 

### Research Questions

We decided to focus on the clinical perspective for evaluation. Before starting database search, the following research questions were posed:

(1) Does surgery alter the gut microbiota composition of a patient and if so, in which kind of extent?
(2) Is the occurrence of an anastomotic leakage related to the composition of a patient’s gut microbiota?
(3) Is the occurrence of a surgical site infection related to the composition of a patient’s gut microbiota?

## 3. Results

### 3.1. Research Question 1: Changes of Gut Microbiota in Surgical Patients

The gut microbiota is rapidly alterable and can be affected by a variety of external factors such as diet and physical activity [31,32,33]. Moreover, it is subject to an inner circadian rhythm [16,34]. Stress events—regardless of psychological or physical origin—can lead to a change of the gut microbiota [35]. Surgery is a massive stress event to the human body, but clinical trials focusing on gut microbiota changes in surgical patients are widely lacking, except for bariatric surgery, which was excluded from this review [36,37]. One potential reason for lack of clinical data is that all patients undergoing surgery receive an intravenous intraoperative or preoperative antibiotic prophylaxis. Both induce a change in the gut microbiota composition and gut immunity making it impossible to see the sole effect of surgery on gut microbiota [13,38]. The consequence of antibiotic treatment is bacterial selection, resulting for example in higher rates of postoperative *Clostridium* infections when antibiotics are administered for several days [22,39,40,41]. Ohigashi et al. showed a significant decrease of bacterial counts comparing pre- and post-operative stool samples of patients, who underwent colorectal surgery due to cancer [42]. All patients received a preoperative oral decontamination (kanamycin and metronidazole) as well as an intraoperative intravenous antibiotic prophylaxis (Cefmetazole). The observation of a bacterial decrease after surgery is confirmed by other clinical trials reporting also an increase of potentially pathogenic bacteria after surgery [29]. Similar observations were reported from rats after application of metronidazole, ampicillin, and kanamycin: Hegde et al. found a significant decrease of bacterial diversity as well as an increase of the relative abundance of *Proteobacteria* and a decrease of the relative abundance of *Bacteroidetes* [43]. The results are plausible as a depletion of bacteria can be expected after application of broad-spectrum antibiotics. These days, surgery is never done without antibiotic prophylaxis implying that the effect of surgery on postoperative microbiota alteration in clinical trials is always biased by antibiotic treatment.

Despite standard administration of perioperative antibiotics, significant changes of gut microbiota can be observed in surgical patients: bowel obstruction, for example due to colorectal carcinoma, is associated with a change of gut microbiota composition [43,44]. The aforementioned study by Hegde et al. observed that the diversity measured by Shannon Index was higher in rats with bowel obstruction than in rats without. All rats underwent surgery prior to stool sampling, but only one group, the bowel obstruction group, received a silicon band permanently placed in the distal colon causing a bowel obstruction. Furthermore, Hegde et al. also found a significant decrease in the relative abundance of *Firmicutes* and an increase in the relative abundance of *Bacteroidetes* and *Proteobacteria* in rats with bowel obstruction. The alteration of gut microbiota is not explainable by antibiotic treatment as all of the rats (with and without bowel obstruction) received a perioperative antibiotic cocktail consisting of metronidazole, ampicillin, and kanamycin.

Another trial by Jin et al. reported the results of a prospective cohort study evaluating the gut microbiota of colorectal cancer patients with pre- or post-operative ileus compared to patients with no ileus [44]. They found significant differences of diversity measured by Shannon Index and UniFrac in patients with and without ileus. Patients with ileus had lower levels of *Firmicutes* (43% vs. 57%), *Bacteroidetes* (23% vs. 77%), and *Fusobacteria* (20% vs. 80%), but higher levels of *Proteobacteria* (71% vs. 29%) and *Actinobacteria* (61% vs. 39%) compared to patients without ileus. The risk of postoperative ileus was significantly increased when *Faecalibacterium* was depleted preoperatively [44]. The results of the trial by Jin et al. are interesting naming a potentially predictive microbial species for prevention of postoperative ileus, but the study validity is limited due to missing data about antibiotic treatment of included patients. Nevertheless, Faecalibacterium prausnitzii is often described as a key marker for a harmful bacterial dysbiosis [45]. Faecalibacterium is a Gram-positive, anaerobic commensal of the human gut, which is able to produce butyrate by fermentation of dietary fiber [45,46]. Lower abundance of Faecalibacterium prausnitzii, the most common subspecies of Faecalibacterium, is supposed to be associated with a variety of diseases such as Crohn’s disease, colorectal cancer and depression [47].

An animal study by Shin et al., induced postoperative ileus in guinea pigs by a coecal suture showing also a postoperative bacterial dysbiosis and an alteration of β-diversity [48]. They found a significant postoperative decrease of genera Bifidobacterium, Lactobacillus, Bacteroides and Blautia compared to the baseline. Similar to the trial of Jin et al., nothing was reported about antibiotic treatment of animals. The reason for the suggested dysbiosis in ileus appears to be multifactorial and is only partially explored. Complex human intestinal in vitro models emphasize the role of epithelial cells for bacterial overgrowth in ileus [49].

The occurrence of ileus is a potentially life-treating event, and it is well-known that the composition of human microbiota is changing distinctly and long-lasting in case of critical illness as well as in case of long-time hospitalization [50,51,52]. Yeh et al. reported a decreased alpha diversity of gut microbiota as well as a depletion of potentially healthy microbes such as *Faecalibacterium* in critically ill surgical patients [53]. The gut microbiota composition is also suggested to be associated with disease severity, which was for example shown in patients with SARS-CoV-2 infection [54,55]. In case of recovery from illness, there is a relation between the recovery of the human body and the recovery of the gut microbiota, but in many critical ill patients longer-lasting changes of gut microbiota are reported [50,51,54]. In surgical patients, studies representing the relation between disease severity and gut microbiota alteration as well as postoperative gut microbiota recovery are widely lacking.

The interplay between the immune system and the gut microbiota seems to be the reason for gut microbiota alteration in infected patients [22,56,57]. Shimizu et al. reported a decreased abundance of obligate anaerobic bacteria in patients with systemic inflammatory response syndrome emphasizing the hypothesis that infectious pathogens appear to predominantly be facultative anaerobic bacteria [52]. In surgical patients, the selection of facultative anaerobes might also be caused by the oxygen exposure due to open surgery [35]. Studies in patients with refractory colitis being treated by fecal transplantation emphasize the role of the interaction between the immune microenvironment and the gut microbiota [58]. In a healthy gut, the physical contact between intestinal epithelial cell surface and the gut microorganisms is minimized by mucus, antimicrobial proteins and IgA to reduce the inflammatory response [22,56]. The mucosal barrier can be destroyed by a disequilibrium of the human homeostasis [14,29]. Surgery is known to be able to disturb homeostasis [59,60]. Thus, the disturbed local immunity of the gastrointestinal tract leads to susceptibility towards infections. Therefore, it is assumable that surgical procedures lead to a bacterial translocation and a measurable alteration of the gut microbiota composition.

**Figure 1:**
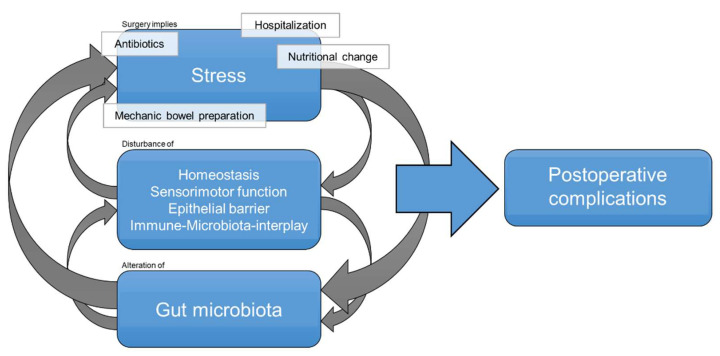
Overview of the relation between surgery and gut microbiota composition.

All in all, it is more than likely that surgery and associated procedures have detrimental consequences for the gut microbiota which can trigger postoperative complications in this vulnerable cohort (Figure 1). Underlying mechanisms are still just partially elucidated, and the individual extent of changes remains widely unclear. Recent publications provide promising microbial biomarkers such as Faecalibacterium prausnitzii, but a high-risk microbial profile for development of postoperative complications is still not defined and should be a target of further research.

### 3.2. Research Question 2: The Relation between the Gut Microbiota and the Development of Anastomotic Leakage

In case of intraoperative necessity of a gastrointestinal anastomosis, one of the most feared postoperative complications in visceral surgery is anastomotic leakage (AL), as it leads to peritonitis, sepsis and even death in case of delayed diagnosis and therapy. AL is defined as a disorder of anastomotic wound healing implying leakage of bowel content into the peritoneal cavity [61]. The clinical severity of AL depends on the amount and quality of leaking fluid. The clinical presentation and the occurrence of AL depends on the performed anastomosis. Higher AL rates were reported for colorectal anastomosis, leaking in almost every fourth to fifth patient [62]. Surgeons are unable to reliably predict occurrence of AL, because the origin of AL is complex. A variety of surgery-related as well as patient-related risk factors were identified to be associated with development of AL (Table 1). AL is also assumed to be related to composition of gut microbiota [26].

#### 3.2.1. Decades Ago: Oral Decontamination to Prevent Anastomotic Leakage

The idea of an impact of gut microbiota on development of AL goes back to the 1980s when Cohen et al. reported an effect of antibiotic bowel preparation on colonic wound healing in rats [69]. In the 1990s, Schardey et al. recommended a preoperative gut decontamination before esophagojejunostomy with regard to their results of a randomized-controlled trial [70]. A cohort study in rectal cancer patients showed a low AL rate of 5.8% and a good safety profile of oral decontamination (polymyxin B, gentamicin, amphotericin B) [20]. Another randomized-controlled trial by Abis et al. failed to show a clear difference of AL rate in colorectal cancer patients with and without oral decontamination (colistin, tobramycin and amphotericin B) [71].

A systematic review, published in 2013, emphasized the role of preoperative decontamination to prevent postoperative AL in gastrointestinal surgery [72]. Meta-analysis of eight randomized-controlled trials emphasized a significant lower rate of postoperative infectious complications after oral decontamination. A further meta-analysis by Rollins et al., published in 2019 and focusing on prevention of AL in elective colorectal surgery, suggested an effect of oral decontamination plus mechanic bowel preparation on AL rate, but lacked to show efficacy of sole oral decontamination [73].

The extent of an individual’s gut microbiota alteration by oral decontamination is broadly unknown. Typical applied antibiotics for oral decontamination are aminoglycosides such as neomycin being primarily effective against Gram-negative bacteria and being not-absorbable after oral administration [72]. The above mentioned oral antibiotics colistin and neomycin are used to eradicate multi-drug resistant *Enterobacteriaceae* prior to fecal transplantation [74,75]. Oral application of colistin and neomycin is known to change gut microbiota distinctly leading to a significantly lower bacterial richness and diversity and showing lower abundance of potentially beneficial genera such as *Bifidobacteria, Roseburia* and *Blautia* [76].

Even if research still suggests an effect of oral decontamination on AL rate, recent recommendations for application of oral antibiotics are restrained due to existing ambiguities [77]. Nevertheless, the results of the studies emphasize the role of the gastrointestinal microbiota for development of AL.

#### 3.2.2. Bacteria Being Potentially Responsible for Development of Anastomotic Leakage

The location of anastomosis is crucial for the expectable spectrum of bacteria in case of postoperative infectious complication. Gram-negative and anaerobic bacteria are more frequently found in patients with colorectal perforation or perforated appendicitis than in patients with gastroduodenal perforations [50]. The lower gastrointestinal tract is typically colonized by a large variety of bacterial genera such as *Bacteroides*, *Clostridia*, *Ruminococci*, *Bifidobacteria* and *Enterococcus*, whereas *Streptococcus* and *Lactobacilli* are more often found in the esophagus and the stomach [78].

A typical commensal being potentially responsible for occurrence of AL is *Enterococcus*. *Enterococci* are Gram-positive, facultative anaerobic bacteria, which are known to be found in infectious diseases, but are also able to protect the gut microbiota by producing bacteriocins [79,80,81]. A subspecies of *Enterococcus, Enterococcus faecalis*, is able to contribute to degradation of collagen and to activate tissue matrix metalloprotease-9 (MMP9) in host intestinal tissue leading to tissue degradation being potentially responsible for AL [82]. Shogan et al. showed a 500-fold increase in the relative abundance of *Enterococci* in the anastomotic tissue of rats [83]. Belmouhand et al. indicated that *Enterococci* were significantly more frequent in drain fluid of patients with AL after pancreaticoduodenectomy [84]. Schmitt et al. reported a spontaneous clustering of patients after pancreas surgery showing a higher abundance of *Akkermansia*, *Enterobacteriaceae* and *Bacteroidales* and a lower abundance of *Lachnospiraceae*, *Prevotella* and *Bacteroides* in patients with postoperative complications [85]. Mima et al. reported results of 256 patients after colorectal carcinoma surgery, the rate of AL was associated with the amount of *Bifidobacteria* and not of *Enterococcus faecalis* [86]. This is interesting as *Bifidobacteria* are also Gram-positive bacteria being often classified as beneficial and health-promoting [87]. Furthermore, recent research suggests a role of *Bifidobacteria* in cancer treatment [88]. Van Praagh et al. reported a higher abundance of *Lachnospiraceae* in anastomotic tissue of patients with AL compared to patients without AL after colorectal surgery [89,90]. *Lachnospiraceae* are also postulated to be beneficial as they are able to produce butyrate and other short-chain fatty acids being often reported to promote health [91,92].

Focusing on the search for a preoperative microbial risk profile for development of AL, Palmisano et al. compared the results of pre- and post-operative stool samples of colorectal cancer patients [93]. They found an enrichment of *Acinetobacter Iwoffii*, *Acinetobacter jhonsonii* and *Hafnia alvei* and a depletion of *Barnesiella intestinihominis* and *Faecalibacterium prausnitzii* in patients with later diagnosis of AL. *Acinetobacter Iwoffii* and *Acinetobacter jhonsonii* are subspecies of the genus *Acinetobacter* belonging to the phylum *Proteobacteria*. Acinetobacter are Gram-negative bacteria considered as commensals of the skin, but also being commonly found in case of hospital-acquired infections [94]. Nowadays, *Hafnia alvei* belongs also to the phylum *Proteobacteria* and is a Gram-negative commensal of the gastrointestinal tract. *Hafnia alvei* is a rare cause of infection being mostly found in immunocompromised patients [95]. *Barnesiella intestinihominis* is a Gram-negative bacterium, which can be found in the human feces [96]. Only a few publications deal with this subspecies of *Bacteroidetes* reporting an impact of *Barnesiella intestinihominis* on the efficacy of chemotherapies [97].

At this point, summing up the above-mentioned trials, it is questionable whether single bacterial subspecies are responsible for development of AL. To date, preoperative stool sampling to detect patients at high risk for development of AL appears to be not constructive as recent data is too inhomogeneous to identify a harmful bacterial composition of the gut. Rather, it appears that occurrence of AL is an interplay of several factors and not only of a surgery-driven or even more an antibiotic-driven dysbiosis.

### 3.3. Research Question 3: The Relation between the Gut Microbiota and the Development of Surgical Site Infections

Surgical site infections (SSIs) are diagnosed in up to 25% of patients after visceral surgery making it one of the most frequent hospital-acquired infections [3,10,11]. The highest risk of SSI can be found in colorectal surgery being explained by the intraoperative contact to the non-sterile intestinal lumen [2]. According to Horan et al., SSI are separated into superficial SSI, deep incisional SSI and organ/space SSI, which are occurring within 30 days after surgery without implantation of a foreign body and within 1 year after implantation of a foreign body [98]. Superficial SSIs are defined as a skin or subcutaneous tissues involving infection after surgical incision showing at least one of the following: purulent drainage, positive blood culture of aseptically obtained fluid or tissue from the incision, signs of infection (swelling, pain, redness, heat). Deep incisional SSI involves deep soft tissues such as fascial and muscle layers and is accompanied by purulent drainage or abscess, fever, pain, and tenderness unless site is culture negative. Organ/space SSI are defined by abscess and purulent drainage or positive blood culture of aseptically obtained fluid or tissue from the organ/space [11].

The origin of SSI is complex as a variety of procedure-related as well as patient-related risk factors were identified to be associated [11,12,13,99]. Most of the above-mentioned risk factors for development of AI are also relevant for development of SSI (Table 1). The source of microorganisms being involved in SSI originate from the skin, the surrounding tissue or from the gut [2]. Overall, *Staphylococcus aureus* is one of the most common microbes being found in SSI, followed by typical gut commensals such as *Escherichia coli* and *Enterococcus faecalis* [2,100]. In visceral surgery, Gram negative bacilli as well as facultative anaerobes are the most common origin of SSI [4,10,11,101,102,103]. Most of the pathogens are normal inhabitants of the human body making it debatable why some patients develop SSI and others not. As mentioned above, the reason might be a disequilibrium of microbial commensals causing a destruction of the inner barrier against potentially pathogenic bacteria promoting bacterial translocation [22,56].

Several reviews indicate an impact of gut microbiota on development of SSI, but valid data of clinical trials focusing on the relation between SSI and gut microbiota are widely lacking [29,81,104]. Velasco et al. showed an improvement of ear wound healing in mice after gut microbiome transplantation [105]. They found a positive correlation of wound healing with the phylum *Firmicutes* (order *Clostridiales* and *Lactobacillales*) and a negative correlation with the phylum *Verrucomicrobia* and *Proteobacteria* (order *Burkholderiales*). Nevertheless, the transferability of animal studies is discussible as rodents have a different skin structure and another course of wound healing [106].

Clinical trials suggested that application of probiotics/synbiotics might be able to lower infectious complications after surgery, but the validity of data is limited, the results are inhomogeneous and the definition of SSI is not consistent [29]. In a randomized-controlled trial evaluating application of synbiotics before elective abdominal surgery, no significant differences between the synbiotic and control groups in septic complications and SSI rate were found [107]. Similar to the above-mentioned aspects regarding AL, the application of an oral decontamination and an intravenous antibiotic prophylaxis is strongly suggested to decrease SSI rates, but the amount of bacterial change remains unclear [20,72,73,108,109].

Future research has to focus on the relation between gut microbiota and development of SSI. The interplay between the host and the gut microbiota emphasizes a role of gut microbiota composition for the occurrence of SSI, but due to the lack of clinical data it is not possible to draw further conclusions or even to identify gut bacteria, which are responsible for development of SSI.

## 4. Conclusions

The results clearly indicate that surgery alters patients’ gut microbiota composition, but the extent of alteration appears to be widely unclear. The occurrence of AL is highly suggested to be related to the gut microbiota composition. Potentially beneficial bacteria in healthy subjects might be able to be pathogens in surgical patients provoking AL, but the underlying mechanisms are just rudimentarily elucidated. SSI are also assumed to be related to the gut microbiota, but valid clinical data are lacking.

Overall, the results indicate the complexity of understanding the gut microbiota role in postoperative complication development. From a clinical perspective, consideration of patients’ gut microbiota is of high relevance, but the diagnostic value of gut microbiota composition for the development of postoperative complications after visceral surgery remains unclear. Recent research supports the role of gut microbiota for development of postoperative complications, but is not able to identify high risk microbial profile for development of postoperative complications supporting the meaning of a pre- or post-operative stool sampling in prevention of complications. To date, the interaction between bacteria is far above the current understanding of bacteria-driven complications in surgery, and further research has to focus on bacterial-bacterial and bacterial-host interactions in surgical patients to clarify in which constellation commensals become pathogens.

## Figures and Tables

**Table 1:** Factors contributing to or increasing the risk of postoperative anastomotic leakage, adapted from McDermott et al. and other recent research results [26,29,35,61,62,63,64,65,66,67,68].

Surgery-Related	Patient-Related
Duration of surgery > 4 h	Male
Intraoperative blood transfusion	Advanced tumor stage, metastatic disease or local tumor size > 3 cm
Anastomosis of the large intestine	Pre-existing illnesses (vascular, hepatic, pulmonary, renal, diabetes)
Emergency surgery	(Ex)-smoker, alcohol abuse
Absorbable suture	History of radiotherapy or chemotherapy
Double-layer anastomosis	Current sepsis or infectious diseases
Poor viability of anastomosis	Current ileus
Extensive intravenous fluid intraoperatively	Cachexia or malnutrition
Late postoperative enteral nutrition	Obesity
Inexperienced surgeon	(Medicinal) immunosuppression
	Composition of gut microbiota

## Abbreviations

AL: anastomotic leakage, SSI: surgical site infection
